# Semantic Congruence Drives Long-Term Memory and Similarly Affects Neural Retrieval Dynamics in Young and Older Adults

**DOI:** 10.3389/fnagi.2021.683908

**Published:** 2021-09-14

**Authors:** Ricardo J. Alejandro, Pau A. Packard, Tineke K. Steiger, Lluis Fuentemilla, Nico Bunzeck

**Affiliations:** ^1^Department of Psychology, University of Lübeck, Lübeck, Germany; ^2^Department of Experimental Psychology, Ghent University, Ghent, Belgium; ^3^Center for Brain and Cognition, Department of Information and Communication Technologies, Universitat Pompeu Fabra Roc Boronat, Barcelona, Spain; ^4^Cognition and Brain Plasticity Group, Bellvitge Biomedical Research Institute (IDIBELL), Hospitalet de Llobregat, Barcelona, Spain; ^5^Department of Cognition, Development and Educational Psychology, University of Barcelona, Barcelona, Spain; ^6^Institute of Neurosciences, University of Barcelona, Barcelona, Spain; ^7^Center of Brain, Behavior and Metabolism (CBBM), University of Lübeck Ratzeburger Allee, Lübeck, Germany

**Keywords:** aging, ERP, EEG, congruence effect, long-term memory

## Abstract

Learning novel information can be promoted if it is congruent with already stored knowledge. This so-called semantic congruence effect has been broadly studied in healthy young adults with a focus on neural encoding mechanisms. However, the impacts on retrieval, and possible impairments during healthy aging, which is typically associated with changes in declarative long-term memory, remain unclear. To investigate these issues, we used a previously established paradigm in healthy young and older humans with a focus on the neural activity at a final retrieval stage as measured with electroencephalography (EEG). In both age groups, semantic congruence at encoding enhanced subsequent long-term recognition memory of words. Compatible with this observation, semantic congruence led to differences in event-related potentials (ERPs) at retrieval, and this effect was not modulated by age. Specifically, congruence modulated old/new ERPs at a fronto-central (Fz) and left parietal (P3) electrode in a late (400–600 ms) time window, which has previously been associated with recognition memory processes. Importantly, ERPs to old items also correlated with the positive effect of semantic congruence on long-term memory independent of age. Together, our findings suggest that semantic congruence drives subsequent recognition memory across the lifespan through changes in neural retrieval processes.

## Introduction

Learning novel information can be promoted if it is congruent with already stored long-term knowledge (Craik and Tulving, 1975; Hall and Geis, 1980; Atienza et al., 2011; Tse et al., 2011; Packard et al., 2017). In cognitive psychology, this so-called “congruence effect” has been explained through the integration of information into knowledge structures or schemas (see also Piaget, 1952). On the basis of functional imaging studies, including electroencephalography (EEG) and functional magnetic resonance imaging (fMRI), recent work demonstrated that encoding-specific processes play an essential role (see below). However, it remains unclear how semantic congruence during encoding changes retrieval dynamics and whether these processes change during healthy aging, which is known to be characterized by impairments of declarative long-term memory.

In typical experiments on the long-term effects of semantic congruence, a semantic cue, for instance, a word such as “instrument,” predicts the presentation of a target that can be semantically congruent, for instance “guitar,” or incongruent, for instance, “tree” (Packard et al., 2017, 2020). fMRI studies suggest that the long-term memory advantage for congruent items directly relates to a modulation in connectivity between the prefrontal cortex (PFC) and medial temporal lobe (MTL, including the hippocampus; van Kesteren et al., 2010, 2013; Sommer, 2017). According to the “schema-linked interactions between medial prefrontal and medial temporal regions” (SLIMM) model (van Kesteren et al., 2012), the medial prefrontal cortex (mPFC) “resonates” with congruent information and therefore inhibits MTL activity in order to drive semantic integration (see also van Kesteren et al., 2012, 2013, 2014). EEG studies could provide further evidence for encoding specific effects by showing that semantic congruence accelerates the onset of the event-related potentials (ERPs) for successful memory encoding (Packard et al., 2017). Moreover, semantic congruence at encoding leads to differences in ERPs starting at around 400 ms after stimulus onset, as well as theta (4–8 Hz), alpha (8–13 Hz), and beta band (14–20 Hz) oscillations (Packard et al., 2020). Importantly, these congruence-related ERPs predicted increases in memory performance for congruent items, further suggesting that ERPs and neural oscillations underlie the congruence effect (Höltje et al., 2019; Packard et al., 2020).

While little is known about the neural dynamics of congruence-dependent memory retrieval, electrophysiological studies indicate specific correlates of recognition memory. For instance, post-stimulus ERPs during retrieval typically show a more positive deflection for correctly identified “old” items as compared to correctly identified “new” items [i.e., the “ERP Old-New Effect” (Rugg, 1995; Danker et al., 2008)]. Moreover, dual-process models suggest that recognition can be associated with specific details or associations of the encoding episode (i.e., recollection), or the absence of such recollective experience (i.e., familiarity; Krantz et al., 1974; Jacoby and Dallas, 1981; Yonelinas, 2001), and both aspects appear to be linked to different ERP components (Düzel et al., 1997; Curran, 2000). Familiarity-based recognition is typically associated with a midfrontal ERP component peaking between 300 and 500 ms, often labeled the FN400 (Rugg and Curran, 2007; Bridger et al., 2012). Recollection based recognition memory, on the other hand, is associated with later ERP components, typically observed from around 400–800 ms at left parietal electrodes (Sanquist et al., 1980; Düzel et al., 1997; Curran, 2000; Rugg and Curran, 2007; Danker et al., 2008). Additionally, both components were linked to confidence level (sure, unsure) at retrieval: item memory strength is associated with the FN400 and source memory strength is associated with the late positive complex (LPC; Woroch and Gonsalves, 2010; Wynn et al., 2020).

Similar to ERPs, neural oscillations in specific frequency bands, namely theta, alpha, and beta, are thought to be crucial for memory retrieval (Klimesch, 1999; Fell and Axmacher, 2011). Specifically, theta power increases (i.e., theta synchronization) in combination with alpha power decreases (i.e., alpha desynchronization) are associated with enhanced memory performance (Klimesch, 1999; Sauseng et al., 2002; Klimesch et al., 2004). Moreover, the theta frequency band has been linked to successful encoding and retrieval of semantic information (Klimesch et al., 1997; Bastiaansen et al., 2008), with higher amplitudes for recollection than for familiarity (Klimesch et al., 2001). Likewise, the alpha frequency band also seems to play a role for semantic information at encoding and retrieval, as well as for sensory input, expectancy, and attentional processes (Klimesch et al., 1997; Klimesch, 1997, 1999). Other studies, using combined EEG-fMRI, suggest that theta-alpha oscillations bind the hippocampus, PFC, and striatum during recollection (Herweg et al., 2016). Finally, beta oscillations have been linked to thalamocortical coupling during long-term memory retrieval (Staudigl et al., 2012). Together, frontal ERPs as well as theta, alpha, and beta oscillations play a role in memory retrieval but their possible modulation through semantic congruence remains unclear.

Finally, while most previous studies on semantic congruence have focused on younger participants (i.e., 18–35 years), potential age-related changes and associated neural mechanisms need further investigation. While age-related impairments could be expected on the basis of well-described memory deficits in older adults, it is also clear that semantic memory (i.e., long-term memory for facts independent of time and date) is often preserved until old age (Hedden and Gabrieli, 2004). Indeed, we could show a preserved semantic congruence effect in older adults (Packard et al., 2020), which is compatible with others showing a relatively small effect of aging on semantic relatedness and associated memory deficits (Crespo-Garcia et al., 2012). However, congruence-related ERPs and neural oscillations in the theta, alpha, and beta range (at encoding) were less pronounced in older subjects indicating age-related neural changes in the absence of behavioral deficits (Packard et al., 2020).

In this study, we used EEG to investigate the effects of semantic congruence on subsequent long-term memory, retrieval mechanisms, and possible age-related changes. Knowing that semantic congruence promotes long-term memory in both age groups and that at encoding there was an effect of age on the electrophysiological measures (Packard et al., 2020), we hypothesized: (a) a modulation of retrieval specific ERPs as well as theta, alpha, and beta oscillations; and (b) an age-dependent effect on the underlying neural processes (i.e., group differences: young vs. older subjects). Note that the behavioral data have already been published together with encoding specific EEG activity (Packard et al., 2020). In this study, we re-analyzed the behavioral effects and focused on EEG activity at retrieval. We first employed cluster-based permutation analyses that included all EEG electrodes (see below). Following this rather conservative approach, we focused on the fronto-central electrode Fz and left parietal electrode P3 for both ERP and time frequency (TF) analysis (see "Materials and Methods" section) since the retrieval of information is particularly related to prefrontal and parietal activity (Rugg and Curran, 2007; Preston and Eichenbaum, 2013). Moreover, the PFC is one of the brain regions exhibiting pronounced age-related changes in terms of structure and function (Cabeza et al., 2002; Rajah and D’Esposito, 2005; Craik and Grady, 2009).

## Materials and Methods

### Participants

Twenty-four young (15 females, mean age = 22.54, *SD* = 2.83 years) and 26 older human subjects (16 females, mean age = 64.42, *SD* = 6.56 years) participated in this study. As described previously (Packard et al., 2020), all participants were right-handed, had a normal or corrected-to-normal vision (including color vision), and reported no history of neurological or psychiatric disorders, or current medical problems (excluding blood pressure). The cognitive abilities of older participants were assessed using the Montreal Cognitive Assessment (MoCA) version 7 (Nasreddine et al., 2005), where all participants had a score of 22 or higher, which is considered a cut-off value for Mild Cognitive Impairment (MCI; Freitas et al., 2013). For the ERP analysis, two subjects had to be excluded, and for the TF analysis, five subjects had to be excluded for different reasons (see below).

Participants were recruited through local newspaper announcements or the database of the University of Lübeck (Greiner, 2015). All participants signed a written informed consent and received monetary compensation. The study was approved by the local ethical committee of the University of Lübeck, Germany, and in accordance with the Declaration of Helsinki.

### Behavioral Procedures

**Figure 1:**
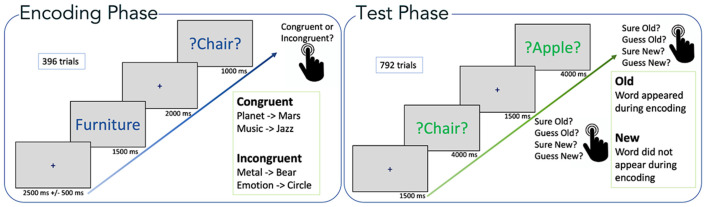
Experimental paradigm. Encoding phase: word pairs are shown (semantic category + target word). Test phase: all target words from the encoding phase were presented randomly intermixed with novel distractors (new words). Figure adapted from Packard et al. (2020).

The experimental paradigm was as described previously (Packard et al., 2020). Briefly, stimuli consisted of 66 categorical six word lists (Packard et al., 2017), selected from category norms (Battig and Montague, 1969; Yoon et al., 2004) translated into German. Each list consisted of the six most typical instances (e.g., Banana, Pear, Grape, Strawberry, Apple, Orange) of a semantic category (e.g., Fruit). The total number of words was 396, all of them were presented in individual encoding trials (see below). The test phase (recognition) included a total of 396 Old-word (all items presented at encoding) and 396 New-word trials (Figure 1).

During the encoding phase, each trial started with a fixation cross in the middle of the screen for a random duration of 2,000–3,000 ms. Subsequently, the name of a semantic category was displayed (white background, blue font) for 1,500 ms, which was followed by a fixation cross for 2,000 ms. Finally, the target word was displayed (white background, green font) for 1,000 ms. During the presentation of the target word, the participants pressed a button to indicate whether the word was congruent (left-hand click) or incongruent (right-hand click) with the semantic category. The condition was congruent if the target word fitted the semantic category (Craik and Tulving, 1975), for example the category “insect” followed by the target word “spider.” The condition was incongruent when the target word did not belong to the semantic category, for example, the category “musical instrument” and the target word “rose.”

The encoding phase lasted about 50 min and included 396 one-word trials, presented in random order. For each category, three words (out of six) belonged to the semantic category (semantically congruent), the remaining three were randomly selected from other categories (semantically incongruent), giving a total of 198 congruent and 198 incongruent stimuli presented during encoding.

Following the encoding phase, participants performed a short (5 min) distraction task, where they had to solve simple arithmetic operations (additions and subtractions). The distraction task prevented the participants from rehearsing the words seen during encoding and prevented recency effects that can contribute to memory.

Finally, the test phase had a duration of approximately 60 min. Here, participants were shown a fixation cross at the beginning of each trial for 1,500 ms, and subsequently, a word (either an Old-word or a New-word item) was displayed (green font, neutral background) for a maximum of 4,000 ms. Participants had to indicate *via* button press whether they judged the word as “sure old,” “guess old,” “guess new,” or “sure new.” The button press determined the end of the trial and it started the presentation of the next trial (i.e., a fixation cross). Participants were allowed to take a break every 50 trials.

### Statistical Analyses of Memory Results

We performed a 2 × 2 repeated measures Analysis of Variance (ANOVA) using Jamovi Version 1.0.8.0 (The jamovi project, 2020), with encoding condition (Congruent vs. Incongruent) as within-subjects factor, and age group (Young vs. Older) as between-subjects factor. Dependent variables were response rates and reaction times (RTs). We included only high-confidence responses in the tests (see below), with α (type I error rate) set to 0.05, $\eta_{p}^{2}$ to estimate effect sizes, and Bayes Factors (BF_10_) to evaluate evidence comparing the alternative hypothesis (1) model to the null (0) model. For effects due to interactions of variables, evidence was evaluated by comparing the BF_10_ of the model with the interaction against the BF_10_ of the model with only the main effects (i.e., BF_10_ interaction model/BF_10_ Main effects model). Note that Bayes factors indicate evidence in favor of the alternative vs. the null hypothesis given the empirical data. Bayes factors between 1 and 3 indicate anecdotal evidence, 3 and 10 moderate evidence, 10 and 30 strong evidence, 30 and 100 very strong evidence, and >100 extreme evidence in favor of the alternative hypothesis. Conversely, 1/3 indicates anecdotal evidence, 1/10–1/3 moderate evidence, 1/30–1/10 strong evidence, 1/100–1/30 very strong evidence, and <1/100 extreme evidence in favor of the null hypothesis. One indicates no evidence (Schönbrodt and Wagenmakers, 2018; Lakens et al., 2020).

As mentioned in our previous work (Packard et al., 2020), response accuracy during the encoding phase was very high (see Table 1). Although there is a significant difference in congruence judgments driven by age (i.e., younger participants had higher accuracy identifying incongruent items than congruent items, while older participants had similar accuracy for both types of trials, see Table 1, and “Results” section), our analysis of the test phase only included trials correctly identified as congruent or incongruent during encoding (see below). Since older participants are prone to more memory errors while retrieving recently learned information with high confidence (Dodson and Krueger, 2006; Dodson et al., 2007a, b; Chua et al., 2009; Shing et al., 2009), we included only high-confidence responses in the analyses. Specifically, corrected Hit Rates (CHR) were calculated by subtracting the proportion of False Alarm (FA; erroneous “old” response to a “new” item) responses from the proportion of hits (correct “old” responses to old words). Only high-confidence responses were included in the calculation of the CHR. Note that FAs could not be classified as congruent and incongruent, because they corresponded to words not presented during encoding.

**Table 1:** Proportion of correct responses and standard deviations (in brackets) during the encoding phase.

Age group	Trial type	Accuracy (%)
Younger	Congruent	91.6 (0.07)
	Incongruent	94.8 (0.06)
Older	Congruent	93.9 (0.04)
	Incongruent	95.2 (0.03)

*Post hoct*-tests were used to evaluate significant interactions detected in the ANOVAs. The Bonferroni correction was used to account for multiple comparisons, lowering the significance level according to the amount of *post hoc* tests performed for each ANOVA. Tests which did not reach the Bonferroni-adjusted significance levels are stated as non-significant.

### EEG Recordings and Analyses

As described in our previous study (Packard et al., 2020), EEG activity during retrieval was acquired using BrainAmp amplifiers, an EasyCap system (BrainProducts GmbH, Munich, Germany), and BrainVision Recorder (version 1.03.0003). We used 32 standard active scalp electrodes, and four electrodes for monitoring vertical and horizontal eye movement (VEOG and HEOG). Electrode impedances were maintained under 20 kΩ. Electrode FCz was used as reference and AFz served as a ground electrode. Data were re-referenced offline to electrode Oz, since re-referencing to the average, although convenient, can alter or suppress the representations of effects with a broad scalp distribution. Furthermore, the average reference delivers waveforms and scalp distributions dependent on the study-specific electrode locations, making it difficult to compare results across studies; it is therefore preferably recommended for high-density montages (Dien, 1998; Luck, 2005). On the other hand, electrode Oz is sensitive to brain activity, but it is located far from the zone of interest (frontal), being thus a suitable reference to measure the full amplitude of the effect in frontal areas and avoid channel distortion (Luck, 2005).

The sampling rate for data acquisition was 500 Hz. The recordings were high-pass (0.1 Hz) and low-pass (240 Hz) filtered online. The open-source EEGLAB (Delorme and Makeig, 2004) toolbox (version 2019), under a customized MATLAB (version R2019b; The MathWorks) environment, was used for preprocessing the EEG data offline.

All trials were limited to a length of 800 ms post-stimulus for epoching. This restriction was necessary since the response to a word ended the trial with the disappearance of the word and the presentation of a fixation cross (i.e., starting the next trial, see “Materials and Methods” section). A longer trial duration could cause the trials from earlier responders (<800 ms) to capture the brain responses to the presentation of the stimulus of the subsequent trial. All trials were epoched accordingly and downsampled to 125 Hz, the latter to reduce computation time, file sizes, and file reading/writing time, without significant loss of information (Seth, 2010; Cohen, 2014). Trials with amplitudes exceeding 100 μV were rejected offline as they were considered artifacts. Eye blinking, saccades, heart beating, and muscle movement artifacts were identified with Independent Component Analysis (ICA; Makeig et al., 1996; Jung et al., 2000; Delorme and Makeig, 2004), implemented in EEGLAB (Makeig et al., 1996; Jung et al., 2000). The artifactual components were selected by visual inspection of scalp maps (head topographies), power spectrum, and ERP plots, and were later removed from the data.

After cluster-based permutation analyses that included all electrodes (see below), *post hoc*, we focused on the fronto-central electrodes Fz and P3 (both ERPs and TF, see “Introduction” section), based on previous studies investigating retrieval dynamics (Wilding and Rugg, 1996; Curran, 2000; Rugg and Curran, 2007; Diana et al., 2011).

### ERP Analysis

The ERP analysis was in accordance with our previous work (Packard et al., 2020). Here, data were low-pass filtered offline using the recommended windowed-sinc FIR filter (Widmann et al., 2015), with a Hamming window, the cut-off frequency at 40 Hz, filter order at 166, implemented in EEGLAB, with no additional high-pass filtering, since the online high-pass filtering was considered sufficient. We analyzed the ERPs by extracting event-locked EEG epochs of 900 ms, starting 100 ms before (baseline signal) and ending 800 ms after stimulus onset. Major artifacts, trials with amplifier saturation, and bad channels were visually identified and removed (maximum four channels, mean = 0.76). ICA (Makeig et al., 1996; Delorme and Makeig, 2004) was performed and finally, bad channels were interpolated. Otherwise, the preprocessing was performed as described in section “EEG Recordings and Analyses”.

For our EEG-data analysis, we focused on those trials that were: (a) correctly classified during encoding as congruent or incongruent; and (b) correctly classified in the test phase as old-word with high-confidence or correctly rejected as new items with high confidence. All other trials (FA, etc.) were not further analyzed. Trial numbers per condition for both the ERP and TF analysis are shown in Table 2. The difference in trial numbers between ERP and TF analysis is due to slightly different preprocessing routines (see “Materials and Methods” section).

**Table 2:** Average trial numbers and standard deviations (in brackets) for EEG Analyses.

Age group	Trial type	Rate	ERP analysis number of trials	TF analysis number of trials
Younger	Sure Congruent Hit	0.63 (0.16)	95.87 (30.84)	100.14 (32.08)
	Sure Incongruent Hit	0.38 (0.17)	58.83 (26.29)	60.95 (26.77)
	Sure False Alarm	0.04 (0.03)	12.09 (10.49)	10.52 (7.61)
	Sure Correct Rejection	0.59 (0.21)	104.09 (67.74)	105.36 (73.24)
Older	Sure Congruent Hit	0.63 (0.19)	102.16 (29.82)	98.14 (27.13)
	Sure Incongruent Hit	0.41 (0.19)	65.00 (29.04)	57.68 (22.25)
	Sure False Alarm	0.06 (0.08)	24.80 (34.80)	16.64 (29.45)
	Sure Correct Rejection	0.79 (0.17)	42.91 (43.13)	51.90 (47.63)

One young and one older participant had to be excluded from the analysis due to excessively noisy data, or since they were regarded as behavioral or electric potential outliers as compared to their age group [identified with Jamovi (The jamovi project, 2020) using a step of 1.5× Interquartile Range]. Therefore, the number of included subjects was not identical to our previous work (Packard et al., 2020), in which we analyzed EEG data from the encoding phase. Here, for the ERP analysis, we included 25 old and 23 younger subjects. Fieldtrip (Oostenveld et al., 2011) and customized MATLAB scripts were used for statistical data analysis *via* a two-tailed non-parametric cluster-based permutation test (Maris and Oostenveld, 2007) to identify differences between the conditions (congruent vs. incongruent trials). The test included all time points between 0 and 800 ms at 27 (out of 28) scalp electrodes, the reference electrode Oz was not considered for the analysis since its activity was canceled out during the re-referencing pre-processing. For every sample (every channel*time-pair), the conditions were compared using a *t*-test. All the samples scoring higher than a specified threshold (0.05) were selected and grouped into clusters, based on temporal and spatial adjacency. The threshold of 0.05 is not the type I error rate for the statistical test, it is a cut-off value for choosing a sample as a member of a cluster. We chose this threshold in accordance with recommendations (Maris and Oostenveld, 2007) and previous literature (Steiger et al., 2019; Packard et al., 2020). Then the sum of *t*-values for each cluster was calculated to obtain the cluster statistics, and the maximum cluster-level statistics was taken as the test statistic which we used to assess the difference between the conditions.

To calculate the significance probability, we used the Monte Carlo method. Random partitions (random samples are extracted from both conditions and put together in a subset, the remaining samples are placed into another subset) were created and the test statistics described above were calculated on those random partitions. This procedure was repeated 1,000 times to generate a histogram of the test statistics. The *p*-value was then obtained with the proportion of cluster statistics in the random partitions exceeding the one calculated from the observed data. Clusters were formed from samples with *p*-values lower than α (0.05); we considered only effects with at least three significant neighboring channels, based on triangulation. Note that significant results from a cluster-based permutation test provide information for rejecting the null hypothesis (absence of an effect), rather than an explanation of the extent of a cluster, which depends on several factors and requires further interpretation (Maris and Oostenveld, 2007; Maris, 2012; Sassenhagen and Draschkow, 2019).

### Time-Frequency Analysis

For the TF analysis, data were low-pass filtered offline using the recommended windowed-sinc FIR filter (Widmann et al., 2015), with a Hamming window, the cut-off frequency at 35 Hz, filter order at 166, implemented in EEGLAB, with no additional high-pass filtering. Major atypical artifacts, trials with amplifier saturation, and bad channels were visually identified and removed (maximum four channels per participant, mean = 2.34). ICA (Makeig et al., 1996; Delorme and Makeig, 2004) was performed and finally, bad channels were interpolated. Five young and one older participant had to be excluded from the TF analysis due to excessively noisy data, or since they were regarded as behavioral or spectral power outliers as compared to their age group [identified with Jamovi (The jamovi project, 2020) using a step of 1.5× Interquartile Range]. Therefore, the number of included subjects was not identical to our previous work (Packard et al., 2020), in which we analyzed EEG data from the encoding phase. Here, for the TF analysis, we included 25 old and 20 younger subjects. Otherwise, the preprocessing was performed as described in section “EEG Recordings and Analyses”.

The TF decomposition was conducted spanning the frequencies from 2 to 30 Hz, in steps of 0.25 Hz, from 500 ms before stimulus onset to 800 ms after stimulus onset, in steps of 8 ms, convolving each single-trial time series with complex Morlet wavelets (4 cycles). The epoch length was extended (using data reflection) to 2,000 ms pre-stimulus, and to 2,000 ms post-stimulus presentation, to ensure that the time window of interest (−500–800 ms) was not interfered with by edge artifacts (Debener et al., 2005; Herrmann et al., 2005; Cohen, 2014, 2017a,b). The average power was obtained across trials. Baseline correction was applied from 500 ms before stimulus onset to 200 ms before stimulus onset, to facilitate interpretation and statistical analyses, and to avoid post-stimulus activity from being averaged into the baseline estimate as much as possible (Cohen, 2017a). The values thus obtained indicated the change in power as compared to the power during the baseline period, that is, with a scale in dB, a value of 0 would indicate no change with respect to baseline.

In order to identify differences between conditions (congruent vs. incongruent), we ran a two-tailed non-parametric cluster-based permutation test (Maris and Oostenveld, 2007) on the frequency range from 2 Hz to 30 Hz. The test is performed similarly as described for the ERPs (section “ERP Analysis”), the difference is that for TF analysis the spectral dimension is added, the samples are thus channel*frequency*time-triplets.

## Results

### Behavioral Findings

#### Encoding

Accuracy of congruence judgement (correctly identifying congruent items as congruent, and incongruent items as incongruent) during the encoding phase was analyzed in a 2 × 2 repeated measures ANOVA. The analysis showed a main effect of congruence (*F*_(1,46)_ = 24.21, *p* < 0.001, $\eta_{p}^{2}$ = 0.345, BF_10_ = 636.02), no significant main effect of age (*F*_(1,46)_ = 0.94, *p* = 0.34, $\eta_{p}^{2}$ = 0.02, BF_10_ = 0.58), and a significant congruence by age interaction (*F*_(1,46)_ = 4.71, *p* = 0.04, $\eta_{p}^{2}$ = 0.09, BF_10_ = 1.64). Subsequent *post hoc* *t*-tests showed a significant difference in congruence for younger participants (*t* = −4.91, *p* < 0.001), which was absent in older participants (*t* = −1.99, *p* = 0.32). See Table 1 for proportion of correct responses during encoding.

#### Retrieval

##### Accuracy

**Figure 2:**
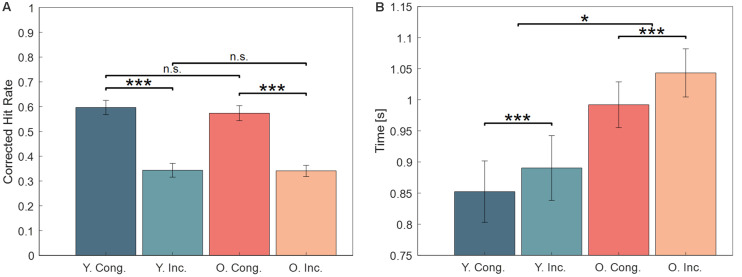
**(A)** Behavioral results. Memory performance was significantly higher for congruent items in both age groups. **(B)** Reaction Times (RT) during recognition of congruent and incongruent items. Correctly responding to incongruent items took longer in both groups, and older participants had overall slower RTs. Error bars show one Standard Error of the Mean (SEM). Abbreviations: Y., young, O., older, Cong., congruent, Inc., incongruent, n.s., not significant. ***Indicates statistical significance at *p* < 0.001 and *p < 0.05.

We analyzed the proportions of high-confidence (Sure) correct responses during the test phase in a 2 × 2 repeated measures ANOVA. The results are shown in Figure 2A, contrasting the congruent and incongruent responses for younger (Y. Cong and Y. Inc.) and for older (O. Cong and O. Inc.) participants. The analysis showed a significant main effect for congruence (*F*_(1,46)_ = 264.09, *p* < 0.001, $\eta_{p}^{2}$ = 0.85, BF_10_ = 5.1 × 10^18^), with a higher CHR for congruent words (mean = 0.57, Standard Error of the Mean (SEM) = 0.02), than for incongruent words (mean = 0.33, SEM = 0.02). The data did not reveal a main effect of age (*F*_(1,46)_ = 0.123, *p* = 0.727, $\eta_{p}^{2}$ = 0.003, BF_10_ = 0.26), and no congruence by age interaction (*F*_(1,46)_ = 0.468, *p* = 0.497, $\eta_{p}^{2}$ = 0.010, BF_10_ = 0.33). This behavioral analysis confirms our previous results (Packard et al., 2020).

##### Reaction Times (RTs)

RTs during retrieval of all correct old responses (high and low confidence) were analyzed in a 2 × 2 repeated measures ANOVA and revealed a main effect of congruence (*F*_(1,46)_ = 21.12, *p* < 0.001, $\eta_{p}^{2}$ = 0.32, BF_10_ = 613.44), a main effect of age (*F*_(1,46)_ = 6.06, *p* = 0.02, $\eta_{p}^{2}$ = 0.12, BF_10_ = 2.31), but no significant congruence by age interaction (*F*_(1,46)_ = 0.75, *p* = 0.39, $\eta_{p}^{2}$ = 0.02, BF_10_ = 0.25). The main effects were driven by overall slower RTs in older subjects and overall faster RTs to congruent items, as shown in Figure 2B. When analyzing only the high confidence old responses, a slightly different pattern emerged: there was a main effect of age (*F*_(1,46)_ = 175, *p* < 0.001, $\eta_{p}^{2}$ = 0.79, BF_10_ = 2.10 × 10^10^), no main effect of congruence (*F*_(1,46)_ = 2.52, *p* = 0.12, $\eta_{p}^{2}$ = 0.05, BF_10_ = 0.65), and no congruence by age interaction (*F*_(1,46)_ = 0.15, *p* = 0.70, $\eta_{p}^{2}$ = 0.003, BF_10_ = 0.31).

### EEG Findings

#### ERP Cluster Analysis

**Figure 3:**
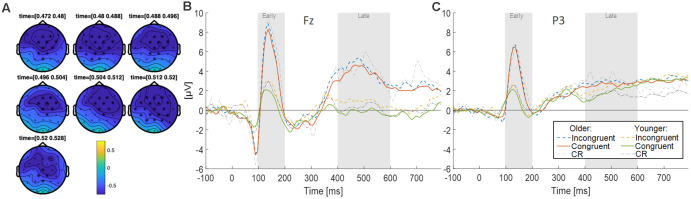
**(A)** Scalp topographies during the time-span of the most prominent clusters due to congruence. Electrodes belonging to the cluster are highlighted with asterisks. **(B)** ERP responses (electrode Fz) for three conditions (Congruent vs. Incongruent vs. Correct Rejections) and age groups (Younger vs. Older adults). **(C)** ERP responses (electrode P3) for three conditions (Congruent vs. Incongruent vs. Correct Rejections) and age groups (Younger vs. Older adults). The analyses for both electrodes focused on an early (100–200 ms) and a late (400–600 ms) time window.

A Monte Carlo cluster-based permutation test was performed on high-confidence responses for correctly recognized old congruent (sure hits) vs. old incongruent words (sure hits), of young and older participants grouped together, from 0 ms to 800 ms after word onset (i.e., main effect of congruence on old responses). The analysis revealed a significant difference between conditions (*p* = 0.004), that appears to be emphasized approximately in the time window from 450 to 550 ms, with a mainly frontal topography but also including central and parietal electrodes (see Figure 3A). The contrast for the main effect age, i.e., young vs. older subjects collapsed across congruent and incongruent sure hits, did not reveal any significant effects. The interaction of congruence and age, as quantified by old congruent minus old incongruent in young participants vs. old congruent minus old incongruent in older participants, also did not reveal any significant results.

### ERP Analyses

#### Effects of Congruence on Old/New Differences at Fz

**Figure 4:**
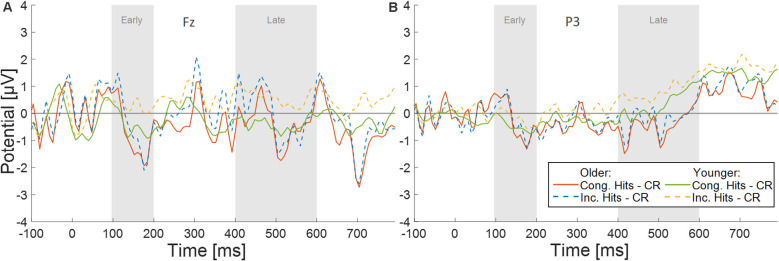
Difference waves for both conditions (Congruent Hits minus Correct Rejection, and Incongruent Hits minus Correct Rejection) for both age groups (Younger and Older participants) for electrodes **(A)** Fz, and **(B)** P3.

In order to more thoroughly characterize the ERPs with respect to typical old/new retrieval dynamics, we studied the waveforms recorded by the electrode Fz and subsequently P3 (see below; Figures 3B,C). After visual inspection of the ERPs, we focused our analysis on an early (100–200 ms) and a late (400–600 ms) time window. Since retrieval effects are often characterized by ERP differences to correctly identified old (hits) vs. new items (correct rejections), we computed difference waves for old congruent vs. new items, and old incongruent vs. new items at Fz for both age groups (see Figure 4A). The averaged old/new differences in both time windows were analyzed separately with 2 × 2 ANOVAs with the factors age and congruence.

The early time window did not show a significant main effect of congruence (*F*_(1,46)_ = 2.85, *p* = 0.10, $\eta_{p}^{2}$ = 0.06, BF_10_ = 0.64), there was no significant main effect of age (*F*_(1,46)_ = 0.18, *p* = 0.68, $\eta_{p}^{2}$ = 0.004, BF_10_ = 0.53), and no congruence by age interaction (*F*_(1,46)_ = 1.87, *p* = 0.18, $\eta_{p}^{2}$ = 0.04, BF_10_ = 0.62). The late time window revealed a significant main effect of congruence (*F*_(1,46)_ = 7.19, *p* = 0.01, $\eta_{p}^{2}$ = 0.14, BF_10_ = 3.33), but no significant main effect of age (*F*_(1,46)_ = 0.01, *p* = 0.91, $\eta_{p}^{2}$ = 0.000, BF_10_ = 0.47), and no significant interaction of congruence by age (*F*_(1,46)_ = 2.67, *p* = 0.11, $\eta_{p}^{2}$ = , BF_10_ = 0.81). The main effect of congruence was driven by more negative deflections in the old/new difference wave to congruent items (Figure 4A).

#### Effects of Congruence on Old/New Differences at P3

Similar to Fz, averaged old/new differences from both time windows at P3 (see Figure 4B) were analyzed separately with 2 × 2 ANOVAs with the factors age and congruence. The early time window did not show a significant main effect of congruence (*F*_(1,46)_ = 0.68, *p* = 0.41, $\eta_{p}^{2}$ = 0.02, BF_10_ = 0.27), no significant main effect of age (*F*_(1,46)_ = 0.83, *p* = 0.37, $\eta_{p}^{2}$ = 0.02, BF_10_ = 0.62), and no congruence by age interaction (*F*_(1,46)_ = 1.31, *p* = 0.26, $\eta_{p}^{2}$ = 0.03, BF_10_ = 0.48). The late time window showed a significant main effect of congruence (*F*_(1,46)_ = 5.36, *p* = 0.03, $\eta_{p}^{2}$ = 0.10, BF_10_ = 1.54), no significant main effect of age (*F*_(1,46)_ = 2.78, *p* = 0.10, $\eta_{p}^{2}$ = 0.06, BF_10_ = 1.003), and no significant interaction of congruence by age (*F*_(1,46)_ = 0.84, *p* = 0.37, $\eta_{p}^{2}$ = 0.02, BF_10_ = 0.35). The main effect of congruence was driven by more negative deflections in the old/new difference wave to congruent items (Figure 4B).

### TF Cluster Analysis

As with the ERP analysis, a Monte Carlo cluster-based permutation test was performed on the high-confidence responses for correctly recognized old (sure hits) congruent vs. incongruent words (sure hits), comparing changes in spectral power elicited by word cues of young and older participants grouped together, from 0 ms to 800 ms after word onset. The analysis revealed no statistically significant effects (*p* > 0.05). Similarly, the contrast for the main effect of age (i.e., young vs. older subjects collapsed across congruent and incongruent sure hits), and the interaction (i.e., congruent minus incongruent in young participants vs. congruent minus incongruent in older participants), neither revealed any significant effects (*p* > 0.05).

### TF Analysis for Fz

**Figure 5:**
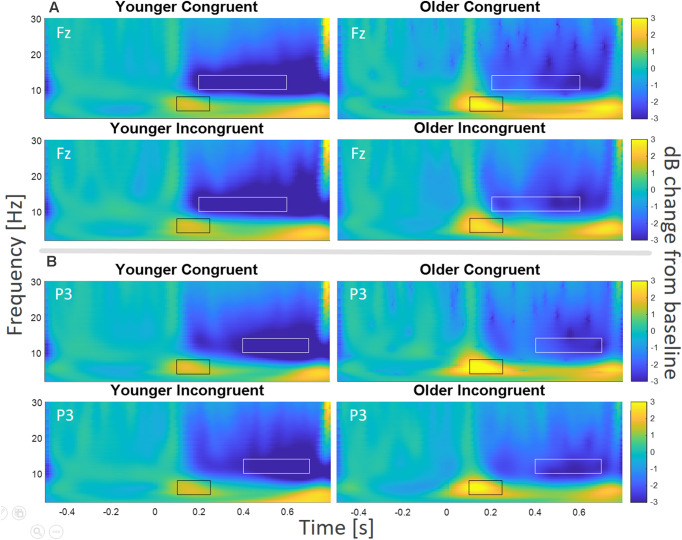
TF Analysis for electrodes **(A)** Fz and **(B)** P3. For Fz, the theta frequency band (black rectangles) was analyzed from 4 to 8 Hz, from 100 to 250 ms, and the alpha frequency band (white rectangles) was analyzed from 10 to 14 Hz, from 200 to 600 ms, for young and older participants. For electrode P3 theta (black rectangles) was analyzed from 4 to 8 Hz, from 100 to 250 ms, and alpha (white rectangles) was analyzed from 10 to 14 Hz, from 400 to 700 ms, for young and older participants.

We followed the rather conservative cluster-based permutation by a specific TF analysis of the electrode Fz. After visual inspection (Figure 5A), we compared the relative change in power for the alpha and theta frequency bands. Specifically, we focused on a time window from 100 to 250 ms, from 4 to 8 Hz for theta (black rectangles in Figure 5A), and on a time window from 200 to 600 ms, from 10 to 14 Hz for alpha (white rectangles in Figure 5A).

The test on the early theta effect revealed a main effect of age (*F*_(1,43)_ = 5.91, *p* = 0.02, $\eta_{p}^{2}$ = 0.12, BF_10_ = 2.35), but no main effect of congruence (*F*_(1,43)_ = 0.03, *p* = 0.859, $\eta_{p}^{2}$ = 0.001, BF_10_ = 0.22), and no congruence by age interaction (*F*_(1,43)_ = 2.85, *p* = 0.098, $\eta_{p}^{2}$ = 0.06, BF_10_ = 1.10). The main effect of age was driven by enhanced theta synchronization for older participants (Figure 5A). The analysis on the later alpha effect showed a significant main effect of age (*F*_(1,43)_ = 3.94, *p* = 0.05, $\eta_{p}^{2}$ = 0.08, BF_10_ = 1.20), but no main effect of congruence (*F*_(1,43)_ = 3.46, *p* = 0.07, $\eta_{p}^{2}$ = 0.08, BF_10_ = 1.04), and no congruence by age interaction (*F*_(1,43)_ = 0.14, *p* = 0.71, $\eta_{p}^{2}$ = 0.003, BF_10_ = 0.33). The main effect of age was driven by lower alpha power for the younger group (see Figure 5A).

### TF Analysis for P3

Compatible with our ERP analysis, we explored TF effects at P3. Visual inspection (see Figure 5B) also revealed an early theta effect (100–250 ms, black rectangles in Figure 5B) and a later alpha effect, which was most pronounced from 400–700 ms (white rectangles in Figure 5B).

The test on the early theta effect revealed a significant main effect of age (*F*_(1,43)_ = 6.65, *p* = 0.01, $\eta_{p}^{2}$ = 0.13, BF_10_ = 3.31), no main effect of congruence (*F*_(1, 43)_ = 0.39, *p* = 0.54, $\eta_{p}^{2}$ = 0.009, BF_10_ = 0.32), and no congruence by age interaction (*F*_(1,43)_ = 0.04, *p* = 0.85, $\eta_{p}^{2}$ = 0.001, BF_10_ = 0.31). Similar to Fz, the main effect of age was driven by enhanced theta synchronization for older participants. The analysis on the later alpha effect showed no significant main effect of age (*F*_(1,43)_ = 2.07, *p* = 0.16, $\eta_{p}^{2}$ = 0.05, BF_10_ = 0.98), no main effect of congruence (*F*_(1,43)_ = 0.98, *p* = 0.33, $\eta_{p}^{2}$ = 0.22, BF_10_ = 0.31), and no congruence by age interaction (*F*_(1,43)_ = 0.82, *p* = 0.37, $\eta_{p}^{2}$ = 0.02, BF_10_ = 0.26).

### Correlations of ERPs and Behavior

**Figure 6:**
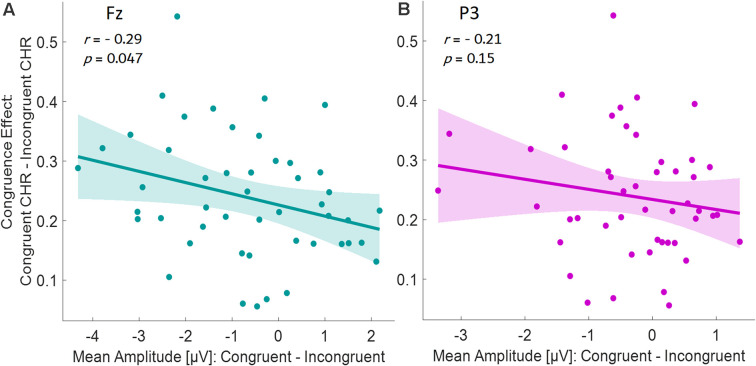
Partial correlations (controlling for age) between the mean ERP amplitudes (Congruent ERP to old items minus Incongruent ERP to old items) as the independent variable, and the memory benefit by congruence (congruent high-confidence CHR minus incongruent high-confidence CHR) as the dependent variable, for the late time window for the electrode **(A)** Fz, and **(B)** P3.

To investigate possible correlates between neural activity specifically to old items and the (congruence-driven) memory benefit, we ran a partial correlation for the late time window at both electrodes (Fz and P3). We used the difference of the mean ERP amplitudes (congruent ERPs to old items minus incongruent ERPs to old items) as the independent variable and the memory benefit by congruence (congruent high-confidence CHR minus incongruent high-confidence CHR) as the dependent variable. For Fz, the correlation was significant in the late time window (see Figure 6A, *r* = −0.29, *p* = 0.047). Here, pronounced memory advantages by congruence were associated with large ERP amplitude differences between congruent and incongruent old items. For P3, no significant correlations could be revealed in the late time window (*r* = −0.21, *p* = 0.15, see Figure 6B).

Note that we focused our correlation analyses only on the late time window since ERPs in the early time window did not show a significant (all *p* > 0.05) modulation by congruence. The same rationale applies to the TF domain, where we also did not observe a modulation of alpha and theta by congruence in any time window.

## Discussion

This study investigated the neural processes underlying the congruence effect with a focus on the retrieval dynamics and possible age-related changes. The behavioral results show that semantic congruence during encoding promotes memory retrieval in both younger and older adults. Compatible with this observation, congruence led to differences in the ERP retrieval old/new effect in a time window from 400 to 600 ms at a frontal and parietal electrode in both age groups. Importantly, the behavioral benefit of semantic congruence correlated with neural activity (ERPs) in this time window, pointing towards a direct relationship. Our findings suggest that semantic congruence drives long-term recognition memory through modulations of retrieval dynamics that are preserved across the lifespan.

At the behavioral level, we confirmed our previous work (Packard et al., 2020), showing that congruent items were better remembered than incongruent items in both age groups. From a general point of view, this is in line with a wealth of studies (Schulman, 1974; Craik and Tulving, 1975; Packard et al., 2017) demonstrating that semantic congruence drives long-term memory. With regard to healthy aging, it is clear that several aspects of long-term memory, including episodic memory, decline as age progresses and this might directly relate to neural degeneration within the prefrontal cortex and medial temporal lobe (Hedden and Gabrieli, 2004; Nyberg et al., 2012; Ofen and Shing, 2013). Therefore, a reduction of the congruence effect could have been expected. However, it has also been suggested that episodic and semantic aspects of long-term memory may show differential age effects with the latter being less pronounced (Ofen and Shing, 2013). In other words, semantic long-term memory is rather stable throughout the lifespan, which may help to explain the absence of age-related differences in the semantic congruence effect in our study. Indeed, and as we have argued before (Packard et al., 2020), this observation is compatible with a previous EEG study (Crespo-Garcia et al., 2012) suggesting a relatively small effect of aging on semantic relatedness and associated memory deficits. However, in some studies, congruence effects were reported to be impaired during healthy aging (Amer et al., 2018, 2019), and this was associated with additional brain activation in older as compared to younger adults (Amer et al., 2019). Since there is only a limited number of published studies on age-related changes in the effect of congruence on long-term memory, further research is needed.

Congruent items were recognized faster than incongruent items (i.e., longer RTs for incongruent words, see Figure 2B) in both age groups, further suggesting that congruent information is retrieved faster and more efficiently than incongruent information. This is in line with the “depth of processing” account (Craik and Tulving, 1975), stating that the integration of congruent information into previous knowledge facilitates subsequent recall since only a portion of the initial information (semantic cue) is needed to extract and complete the representation from memory. Even so, it does not rule out the alternative transfer-appropriate processing account (Morris et al., 1977; Roediger, 2008). Although the RT advantage for congruent items was independent of age, older participants had overall longer RTs at retrieval for both congruent and incongruent items. Such age-dependent and therefore characteristic delays have often been described in the memory literature (Salthouse, 1996; Park et al., 2002; Luo and Craik, 2008). Accordingly, processing speed is notably slower at an older age, and this might be attributable to age-related loss of neural connections (Raz, 2000), changes in neurotransmitter systems (Tromp et al., 2015), impaired neural processing (Salthouse, 2013), and reduced attentional capabilities (Rodrigues and Pandeirada, 2014).

At the neural level, we observed congruence-dependent effects on retrieval-related old/new processes. Specifically, within a late time window (400–600 ms) incongruent items were associated with more negative deflections in the old/new ERPs at a fronto-central (Fz) and parietal electrode (P3). Although EEG has a poor spatial resolution, the activity of Fz presumably reflects activity in underlying frontal brain regions, which fits to a role of the frontal cortex in semantic congruence. The SLIMM model (van Kesteren et al., 2012) suggests that semantically congruent information leads to resonance in the mPFC, which, as a consequence, inhibits MTL activity in order to drive semantic integration. While initial evidence appears to be compatible with SLIMM (van Kesteren et al., 2012, 2013, 2014), others suggest that both the mPFC and MTL together drive semantic integration (McKenzie et al., 2013, 2014; Preston and Eichenbaum, 2013; Gilboa and Marlatte, 2017; Liu et al., 2017; van Kesteren et al., 2020). Although we cannot resolve the precise role of the mPFC and hippocampus on the basis of our EEG data, our findings demonstrate that congruence during encoding modulates subsequent retrieval dynamics at frontal electrodes.

The left parietal old/new ERP effect, on the other hand, may reflect components typically associated with recollection-based recognition memory (Tulving, 1985; Düzel et al., 1997). Such an interpretation is further underlined by the fact that we only included high-confidence responses in our analysis, which are most likely based on recollective experiences rather than familiarity judgments, and the absence of other frontal components indicative of familiarity (Curran, 2000; Rugg and Curran, 2007). Both old/new effects (at Fz and P3) were associated with more positive deflections for incongruent items, and the correlation analysis for the ERP responses to old items showed that memory benefits by congruence directly relates to the ERP differences in the late time window (see Figure 6A). In other words, the more pronounced the semantic congruence effect, the larger the ERP differences for congruent vs. incongruent items. Although this analysis does not allow any causal inferences, it further points towards a direct relationship between subsequent recognition by congruence and neural processes especially in the late time window at retrieval. Since the correlation was observed across all participants when partializing out age, this suggests a common underlying neural mechanism in both age groups.

Although ERPs indexing successful retrieval are often found after 200 ms, earlier retrieval ERPs have also been detected (Bunzeck et al., 2009; Apitz and Bunzeck, 2013). Furthermore, it is interesting to note that although deep-processing has been previously found to increase both late positive and high-confidence retrieval and recollection (Voss and Paller, 2017), here, the items identified as semantically congruent led to greater high-confidence retrieval, but a less pronounced later component. While recollection specific late ERP components may vary in their scalp distribution, they typically have a centro-parietal (and not frontal) topography (Rugg and Curran, 2007; Friedman, 2013). Moreover, the effect of congruence on old/new differences found in parietal (P3), and fronto-central (Fz) locations further supports our interpretation that congruence modulates retrieval through recollection processes. Together, there are apparent differences between previous ERP studies on recognition memory and our work. Therefore, further research is needed to determine more precisely which components of retrieval are increased or facilitated when recognizing items encoded within a congruent semantic context.

For both the early and late time windows, older participants’ ERP responses exhibited greater amplitudes as compared to the younger group. This may be indicative of compensatory mechanisms in order to achieve the same behavioral performance. Indeed, previous work (Cabeza et al., 2002) suggests that during episodic memory retrieval, high performing older subjects (i.e., those that did not differ at the behavioral level from younger subjects) recruit bilateral instead of unilateral prefrontal brain regions. In accordance with this observation, the “Compensation-Related Utilization of Neural Circuits Hypothesis” (CRUNCH; Reuter-Lorenz and Cappell, 2008) suggests that an increase in neural activity in older adults can compensate for age-related cognitive decline if the task is not too demanding. Therefore, recruiting additional or alternative neural resources (as indicated by enhanced ERP amplitudes) might explain the absence of age-related behavioral differences in our study. Along the same lines, a discrepancy between behavioral and physiological responses is not uncommon (Cabeza et al., 1997; Mark and Rugg, 1998; Trott et al., 1999), including studies on semantic memory (Fjell et al., 2005; Duarte et al., 2006). One possibility is that neuroimaging techniques are sensitive enough to detect physiological effects, such as age-related functional or structural changes, which may not necessarily be apparent in behavioral tests. For example, abnormal EEG measures during the early stages of Alzheimer’s disease can predict a severe decline in cognitive functions even when behavioral changes are not yet evident (Helkala et al., 1991).

With regard to neural oscillations, we observed clear alpha and theta power effects during retrieval of congruent and incongruent items that significantly differed between age groups (Figure 5). While alpha power decreases were more pronounced in the younger group, theta power increases were more pronounced in older participants. In general terms, a reciprocal variation in theta and alpha power during retrieval might relate to memory processes (Klimesch, 1999), since their interaction is believed to facilitate information transfer between working memory and long-term memory (Sauseng et al., 2002). Other studies have suggested that theta-alpha oscillations bind the hippocampus, prefrontal cortex, and striatum during recollection (Herweg et al., 2016). Specifically, the suppression of alpha power (desynchronization) is associated with attentional and semantic memory processes during retrieval (Klimesch et al., 1997; Klimesch, 1999). Theta, on the other hand, has been associated with several aspects of encoding and retrieval including the support of associative memory (Herweg et al., 2020). In our task, the processing of sensory information (congruent or incongruent stimuli) requires a semantic evaluation, to extract a meaning and possible associations to prior knowledge.

The observed age affect, as expressed in less decrease of alpha power but enhanced theta power in the older subjects (Figure 5), might indicate age-dependent reductions in attentional processing that might be compensated by higher retrieval efforts in order to achieve the same behavioral performance (Cabeza et al., 2018). In line with our observation, the alpha frequency band reduces with age (Nussbaum, 1997; Rizzo et al., 2017; Knyazeva et al., 2018), at a rate of ~0.08 Hz per year after the age of 60 (Pedley and Miller, 1983), and an increase in alpha desynchronization has been associated with the recruitment of additional attentional resources in participants on early stages of cognitive decline (Deiber et al., 2015). Theta power, on the other hand, appears with a increased power in older subjects, compared to younger controls (Silverman et al., 1955; Nussbaum, 1997; Rizzo et al., 2017). Moreover, older adults with MCI and Alzheimer’s disease patients have both shown a remarkable decrease in alpha power, and an excessive increase in theta power (Jelic et al., 1996; Rossini et al., 2006).

Finally, neural theta, alpha, and beta oscillations did not show significant differences while retrieving semantically congruent vs. incongruent information. This was unexpected since all three frequency bands have previously been associated with learning and memory processes (Fell and Axmacher, 2011; Hanslmayr and Staudigl, 2014; Herweg et al., 2020). Since it is difficult to precisely pinpoint such a null-finding, we refrain from further speculating about the possible reasons.

To conclude, semantic congruence drives subsequent long-term recognition memory across the lifespan, and this effect could be related to neural activity at frontal and left parietal electrodes in a time window that has previously been associated with recollection-based recognition memory. Together with a correlation of ERP responses and behavior, this indicates that neural retrieval processes play a significant role in the memory advantage by semantic congruence. As such, our work gives novel insights into the underlying neurophysiological mechanisms of the semantic congruence effect across the life span.

## Data Availability Statement

The raw data supporting the conclusions of this article will be made available by the authors upon reasonable request.

## Ethics Statement

The studies involving human participants were reviewed and approved by the Ethics Committee at the University of Lübeck. The patients/participants provided their written informed consent to participate in this study.

## Author Contributions

PP, LF, and NB designed the study. PP and TS collected the data. RA and PP ran the analyses. All authors participated in discussion to interpret the results. RA and NB wrote the article, and all the authors participated in revising it. All authors contributed to the article and approved the submitted version.

## Conflict of Interest

The authors declare that the research was conducted in the absence of any commercial or financial relationships that could be construed as a potential conflict of interest.

## Publisher's Note

All claims expressed in this article are solely those of the authors and do not necessarily represent those of their affiliated organizations, or those of the publisher, the editors and the reviewers. Any product that may be evaluated in this article, or claim that may be made by its manufacturer, is not guaranteed or endorsed by the publisher.
